# Molecular Advances in Abiotic Stress Signaling in Plants: Focus on Atmospheric Stressors

**DOI:** 10.3390/ijms26051878

**Published:** 2025-02-22

**Authors:** Mateusz Labudda, Philippe Jeandet

**Affiliations:** 1 Department of Biochemistry and Microbiology, Institute of Biology, Warsaw University of Life Sciences—SGGW, Nowoursynowska 159, 02-776 Warsaw, Poland; 2 Research Unit “Induced Resistance and Plant Bioprotection”, UPRES EA 4707, Department of Biology and Biochemistry, Faculty of Sciences, University of Reims, CEDEX 02, P.O. Box 1039, 51687 Reims, France; philippe.jeandet@univ-reims.fr

## 1. Introduction

Plants face many abiotic stresses that significantly impact their growth and survival, requiring intricate mechanisms in order to adapt and thrive. These responses occur at multiple levels of plant organization, beginning with changes in biochemical processes such as respiration, photosynthesis, and transpiration, which form the basis of stress adaptation. On a broader scale, these biochemical alterations lead to morphological and anatomical changes in plant organs, thereby enabling improved resilience to environmental challenges. Underlying these adaptations is a sophisticated molecular signaling network that acts as an early warning system, allowing the plants to perceive and respond to external abiotic stimuli effectively. This signaling machinery integrates various molecular pathways to initiate appropriate physiological and structural changes. Understanding the interplay between molecular signals and plant responses provides valuable insights into the complex nature of abiotic stress tolerance. Such knowledge is crucial for developing strategies to enhance crop resilience in the face of global environmental changes [1,2,3,4].

This Special Issue presents a collection of 12 original and review articles that explore the key thematic areas highlighted within this SI.

## 2. Experimental Articles

*Crocus sativus* L., a plant renowned for its cultivation of saffron—the most expensive spice in the world—was examined in order to evaluate the effects of pre-sowing treatments on plant performance and saffron quality [5]. The treatments included cold plasma (CP), vacuum, and electromagnetic field (EMF) applications, influencing sprouting kinetics, growth parameters, trichome density, and secondary metabolite content. CP treatment was found to negatively affect plant growth and metabolite content, while EMF treatment notably increased flower length and levels of various metabolites. Although vacuum treatment improved germination uniformity, it had minimal impact on stigma compounds compared to both CP and EMF. Among the treatments, EMF was the most effective at enhancing secondary metabolites, significantly raising the concentrations of crocin, picrocrocin, and safranal. While all treatments preserved corm viability, they induced stressor-specific alterations in plant traits. EMF treatment shows promise for improving saffron quality by enhancing the concentrations of bioactive compounds.

During photosynthesis, reactive oxygen species (ROS) such as hydrogen peroxide (H_2_O_2_) and singlet oxygen (^1^O_2_) are generated, with ^1^O_2_ playing a crucial role in signaling through its reaction products, including lipid peroxides and reactive electrophile species (RES). Pancheri et al. [6] investigated the function of ROS in the high light (HL) acclimation of *Chlamydomonas reinhardtii*, focusing specifically on GreenCut2 genes that are unique to photosynthetic organisms. RNA sequencing analyses revealed that HL significantly influenced the expression of 131 GreenCut2 genes, with more than half of the HL-upregulated genes also responding to RES. Key upregulated genes included *RBCS1*, *PSBS1*, and *LHCSR1*, whereas downregulated genes comprised *CAO1*, *MDH2*, and *PGM4*. The impact of H_2_O_2_ and *β*-cyclocitral on gene expression was found to be limited, underscoring the specific role of ^1^O_2_ in HL responses. Signaling by ^1^O_2_ under HL conditions enhances photoprotection and carbon assimilation while downregulating primary metabolic pathways. These findings indicate that RES-mediated signaling is essential for photosynthetic acclimation to HL conditions.

Selenium nanoparticles (SeNPs) and melatonin (MT), both recognized as bio-stimulants, significantly influence the growth of plants. However, the combined effects of these treatments had not been previously reported. In an article by Kang et al. [7], two melon cultivars were treated with SeNPs, MT, and a combination of both, after which their physiological and biochemical responses were analyzed. While individual and combined treatments did not yield significant changes in plant height or stem diameter, both SeNPs and MT notably increased levels of soluble sugars (by 6–63%) and sucrose (by 11–88%), as well as enhanced the activity of sucrose phosphate synthase (by 171–237%) in leaves. Additionally, there were significant increases in key enzymes and mRNA levels associated with the phenylpropanoid metabolism pathway. Notably, the combined treatment was more effective than either individual treatment, resulting in higher activities of antioxidant enzymes, including superoxide dismutase (SOD), catalase (CAT), and peroxidase (POD). These findings suggest that SeNPs and MT can enhance melon seedling growth by improving carbohydrate metabolism, phenylpropanoid activity, and antioxidant capacity.

The mechanisms through which MT safeguards plants against cadmium (Cd) toxicity are still not fully understood, particularly at the molecular level. Lee et al. [8] successfully identified and cloned a novel serotonin N-acetyltransferase 3 (*SNAT3*) gene in rice (*Oryza sativa*); this gene is a pivotal enzyme involved in MT biosynthesis. *OsSNAT3* converts serotonin and 5-methoxytryptamine into N-acetylserotonin and MT, respectively. The suppression of *OsSNAT3* via RNA interference (RNAi) resulted in reduced MT levels and Cd tolerance in transgenic rice, alongside a decreased expression of endoplasmic reticulum chaperone genes (*BiP3*, *BiP4*, and *BiP5*). In contrast, the overexpression of *OsSNAT3* (referred to as SNAT3-OE) led to elevated MT levels and enhanced stress tolerance to Cd, as demonstrated by improved seedling growth, lower malondialdehyde (MDA) content, and increased chlorophyll levels. Additionally, SNAT3-OE lines displayed a higher expression of *BiP4* compared to wild-type plants. These findings indicate that engineering MT production through OsSNAT3 could boost crop resilience to Cd stress, thereby improving yields in contaminated agricultural fields.

Elevated atmospheric CO_2_ levels play a significant role in global warming and can negatively impact plants’ physiological and biochemical processes, even though CO_2_ is crucial for photosynthesis. Li et al. [9] investigated the physiological and proteomic responses of the tetraploid *Robinia pseudoacacia* to high CO_2_ concentrations (5%). The results showed that elevated CO_2_ levels hindered growth and development, resulted in severe leaf damage, and diminished photosynthetic parameters (*Pn*, *Gs*, *Tr*, and *Ci*), respiration rates, and chlorophyll content. Additionally, chlorophyll fluorescence parameters (*Fm*, *Fv*/*Fm*, *q*P, and *ETR*) showed reductions, while the levels of ROS (H_2_O_2_ and O2^·−^) increased, accompanied by the decreased activity of antioxidant enzymes. The accumulation of ROS and nitric oxide (NO) in guard cells triggered stomatal closure. Proteomic analysis revealed 1652 differentially abundant proteins (DAPs) linked to redox activity, catalytic functions, and various metabolic pathways, including photosynthesis and the biosynthesis of secondary metabolites. These findings offer valuable insights into the adaptation mechanisms of tetraploid *R*. *pseudoacacia* under conditions of high CO_2_ stress.

Transcription factors are essential for regulating plant responses to external stimuli, and the WRKY protein superfamily plays a pivotal role in mediating stress responses to conditions such as cold, heat, salt, drought, and pathogen attacks. The number and functions of WRKY transcription factors have evolved throughout the evolution of angiosperms, with different plant species retaining varying quantities of WRKY family members based on their evolutionary backgrounds. The WRKY family is categorized into three major groups in angiosperms, distinguished by their conserved domains and structural similarities. These transcription factors facilitate plant adaptation to environmental stresses by engaging in ROS and hormone signaling pathways and regulating enzyme activity, stomatal closure, and leaf shrinkage. Wu et al. [10] examined the evolution and functional roles of WRKY factors in angiosperms, particularly emphasizing Magnoliaceae plants. This article offers insights into the functional diversity of the WRKY family and its evolutionary importance. The findings provide theoretical and experimental guidance for understanding the molecular mechanisms underlying environmental stress tolerance in plants.

LESION-SIMULATING DISEASE1 (LSD1) is a vital regulator of cell death in *Arabidopsis thaliana* [11]. Mutations in *LSD1*, leading to the *lsd1* phenotype, result in runaway cell death (RCD) under various stress conditions. The manifestation of the *lsd1* mutant phenotype is influenced by ENHANCED DISEASE SUSCEPTIBILITY 1 (EDS1), PHYTOALEXIN-DEFICIENT 4 (PAD4), and salicylic acid (SA) and ROS signaling pathways. Notably, the conditional RCD phenotype of *lsd1* varies between controlled laboratory settings and field environments, indicating the involvement of different regulatory mechanisms. Transcriptome analysis has identified METACASPASE 8 (MC8) as a potential regulator of RCD associated with *lsd1*. In the *mc8*/*lsd1* double mutant, the RCD phenotype was mitigated under UV radiation, mirroring the outcomes observed in the double mutants *lsd1*/*eds1* and *lsd1*/*pad4*. This study highlights the positive role of MC8 in the EDS1- and PAD4-dependent regulation of cell death in the absence of LSD1 function. A working model has been proposed, positioning MC8 as an essential component of the LSD1-EDS1-PAD4 regulatory hub. These findings provide new insights into the molecular mechanisms governing plant stress-induced cell death [11].

OsBBTI5, a member of the Bowman–Birk inhibitor (BBI) family, is involved in the plant’s stress responses and has been shown to bind specifically to the salt stress-related gene *OsAPX2*. Lin et al. [12] explored the role of the *OsBBTI5* gene in enhancing salt tolerance in rice by employing RNAi to inhibit its expression selectively. Transgenic *OsBBTI5*-RNAi plants demonstrated altered responses to brassinosteroid and gibberellin (GA) treatments, exhibiting reduced growth under high concentrations of GA3. When subjected to salt stress (40–60 mM NaCl), these plants showed increased activities of POD and SOD, along with a decreased MDA content, indicating enhanced stress tolerance. Transcriptomic analysis revealed the significant upregulation of photosynthesis-related genes in transgenic plants compared to wild-type plants under salt stress conditions. The findings suggest that *OsBBTI5* plays a role in the brassinosteroid signaling pathway and interacts with *OsAPX2* to improve stress tolerance. This study offers novel insights into the molecular mechanisms underlying salt tolerance in rice.

## 3. Review Articles

Green leaf volatiles (GLVs) have traditionally been linked to protecting plants from pests and pathogens. However, recent evidence suggests that GLVs also play a significant role in helping plants defend against abiotic stresses such as heat, cold, drought, light, and salinity. Although the molecular mechanisms behind this protective function remain largely unclear, it is evident that the production of GLVs is closely connected to the physical damage caused by these stresses. This damage acts as a primary trigger for GLV synthesis. Engelberth [13] summarized current knowledge regarding GLVs in relation to abiotic stress and proposed a model for their multifunctionality. GLVs appear to mediate plant responses to various stresses, underscoring their broader significance in promoting plant resilience. Further research is necessary to clarify the molecular pathways involved in the stress protection provided by GLVs.

Ultraviolet (UV) radiation presents a significant challenge by causing cellular damage, necessitating robust DNA repair mechanisms supported by histone acetyltransferases (HATs). HATs are vital in regulating chromatin structure and gene expression, facilitating chromatin relaxation and transcriptional activation, which are essential for development and stress responses. Boycheva et al. [14] examined the function of HATs in photomorphogenesis, chromatin remodeling, and gene regulation, emphasizing their significance in light responses and stress adaptation. They underscored the necessity for further research into individual HAT family members and their interactions with other epigenetic factors. Advanced genomic and genome-editing technologies offer promising avenues for enhancing crop resilience and productivity through the targeted modulation of HAT activities.

Drought stress poses a significant challenge to agricultural productivity due to its increasing frequency and intensity and the extensive losses it incurs. Sorghum (*Sorghum bicolor*), a C4 plant, exhibits a range of morphological, physiological, and biochemical adaptations that enable it to thrive in arid conditions [15]. These adaptations encompass improved water uptake, minimized water loss, osmotic potential adjustments, the scavenging of ROS, and the heightened activity of antioxidant enzymes. Moreover, specific genes in sorghum show downregulation in response to drought stress. Liu et al. [15] presented the mechanisms underlying sorghum’s drought tolerance, focusing on its morphological traits, physiological processes, and the identification of functional genes. Implementing modern biotechnological and molecular strategies is essential for enhancing sorghum’s resistance to drought.

Atmospheric stressors such as CO_2_, NO_x_, sulfur compounds, extreme temperatures, ozone, UV-B radiation, and acid rain significantly impact plants, particularly those of agricultural importance. Although NO and hydrogen sulfide (H_2_S) were once viewed solely as toxic pollutants, they are now recognized as vital signaling molecules in plant stress responses [16]. These gasotransmitters enhance enzymatic and non-enzymatic antioxidant systems, strengthening the plant’s defense mechanisms. They play crucial roles in alleviating the effects of various environmental stresses, including those posed by atmospheric conditions. Corpas [16] described the endogenous metabolism of NO and H_2_S within plant cells and underscored their significance in stress signaling pathways. Additionally, the potential of applying NO and H_2_S exogenously to enhance crop resilience has been discussed.

## 4. Conclusions

Research into plant responses to abiotic stress reveals a complex network of molecular, physiological, and biochemical mechanisms essential for adaptation and resilience. Studies have highlighted the promise of innovative treatments—such as EMF, SeNPs, and MT—in enhancing stress tolerance and boosting secondary metabolite production in crops like saffron, melon, and rice. Similarly, investigations into key genes, including *OsSNAT3* and *OsBBTI5*, have demonstrated their roles in stress mitigation by activating crucial biochemical pathways and stress signaling. Additional insights into transcription factors like WRKY and signaling molecules such as NO and H_2_S have emphasized their importance in regulating adaptive responses. Mechanistic studies on ROS and histone acetyltransferases shed light on the plant’s capacity for stress signaling and chromatin-level regulation under HL and UV stress conditions. Future research should aim to explore these molecular pathways in more depth, advance genome-editing technologies, and utilize these insights to develop sustainable agricultural solutions that enhance crop resilience in the face of a rapidly changing climate.

## Data Availability

Not applicable.
